# Clinical, Neuropathic, and Sudomotor Correlates of Orthostatic Hypotension in Type 2 Diabetes: A Cross-Sectional Study

**DOI:** 10.3390/healthcare14111515

**Published:** 2026-05-29

**Authors:** Bianca Iliescu, Andreea Herascu, Laura Gaita, Vlad Florian Avram, Adina Braha, Bogdan Timar

**Affiliations:** 1 Doctoral School of Medicine, “Victor Babes” University of Medicine and Pharmacy, 300041 Timisoara, Romania; bianca.iliescu@umft.ro; 2 Sebes Municipal Hospital, 515800 Sebes, Romania; 3 Centre for Molecular Research in Nephrology and Vascular Disease, “Victor Babes” University of Medicine and Pharmacy, 300041 Timisoara, Romania; gaita.laura@umft.ro (L.G.); avram.vlad@umft.ro (V.F.A.); braha.adina@umft.ro (A.B.); bogdan.timar@umft.ro (B.T.); 4 Department of Diabetes, “Pius Brinzeu” Emergency Hospital, 300736 Timisoara, Romania; 5 Second Department of Internal Medicine, “Victor Babes” University of Medicine and Pharmacy, 300041 Timisoara, Romania

**Keywords:** orthostatic hypotension, type 2 diabetes, autonomic neuropathy, sudomotor dysfunction, diabetic neuropathy

## Abstract

Background/Objectives: Orthostatic hypotension (OH) is a clinically relevant manifestation that may reflect cardiovascular autonomic dysfunction in type 2 diabetes (T2D), yet its correlates remain incompletely characterized. This cross-sectional study evaluated clinical, neuropathic, and sudomotor factors associated with OH and explored balance-related outcomes as secondary analyses. Methods: In this cross-sectional study, 124 adults with T2D aged ≥60 years underwent standardized orthostatic blood pressure testing. Peripheral neuropathy was assessed using the Michigan Neuropathy Screening Instrument (MNSI), and sudomotor function was assessed by electrochemical skin conductance measured with Sudoscan. Balance, mobility, and fear of falling were evaluated as exploratory secondary outcomes. Active antihypertensive treatment was recorded at the time of assessment and considered a potential confounder. Multivariable logistic regression was used to identify factors associated with OH. Results: OH was associated with longer diabetes duration (OR = 1.11/year, *p* = 0.002), higher objective neuropathy severity (MNSI-B; OR = 1.27, *p* = 0.049), and increased urinary albumin-to-creatinine ratio (OR = 1.01, *p* = 0.035). Sudomotor parameters did not differ significantly between OH groups in univariate analyses and were not retained in the final parsimonious model. Exploratory analyses showed no significant univariate differences in balance or fear-of-falling outcomes by OH status. Model discrimination was acceptable (AUC = 0.787), whereas calibration was imperfect according to the Hosmer–Lemeshow test; therefore, model performance should be interpreted as apparent and explanatory rather than predictive. Conclusions: In older adults with T2D, OH was associated with longer disease duration, greater neuropathy burden, and microvascular involvement. Sudoscan-derived measures were not independently associated with OH in this cohort. Because of the cross-sectional design and residual medication confounding, all findings should be interpreted as associations only. These results support routine orthostatic evaluation alongside neuropathy and albuminuria assessment, while predictive modeling requires external validation in larger cohorts.

## 1. Introduction

Falls and balance impairment are frequent and clinically significant complications in adults with type 2 diabetes (T2D), contributing substantially to morbidity, functional decline, and reduced quality of life. While incident falls represent the ultimate clinical outcome, the present study focuses on balance performance and fear of falling as proximal functional correlates that may precede overt fall events. The pooled prevalence of falls in older adults with diabetes is estimated at 29.5% based on a large meta-analysis of 32 studies including over 23,000 participants [1]. Cross-sectional studies report annual fall incidence rates ranging from 19.7% to 36%, consistently exceeding those observed in age-matched non-diabetic populations [2,3].

Multiple factors have been implicated in the increased risk of falls among individuals with T2D, including diabetic peripheral neuropathy (DPN), balance impairment, advanced age, female sex, depression, cognitive dysfunction, reduced muscle strength, and gait abnormalities [1,3,4,5,6,7]. Among these, DPN is a well-established determinant of postural instability, with increasing neuropathy severity associated with poorer balance performance and a higher likelihood of falls. Consequently, current clinical guidelines, including those of the American Diabetes Association (ADA), recommend routine screening for peripheral neuropathy and fall risk in individuals with diabetes, particularly in older adults [6,8]. Small-fiber autonomic dysfunction may also contribute to impaired blood pressure regulation and postural control, thereby linking sudomotor abnormalities with OH and downstream balance deficits.

However, beyond somatic neuropathy, autonomic nervous system involvement represents a critical but often under-recognized contributor to diabetes-related morbidity.

Diabetic autonomic neuropathy (DAN) is a heterogeneous disorder characterized by impaired autonomic regulation affecting multiple organ systems, including cardiovascular, gastrointestinal, genitourinary, and sudomotor function, after exclusion of other etiologies [8,9,10]. Cardiovascular autonomic neuropathy (CAN) is the most extensively studied form of DAN and is associated with adverse outcomes such as exercise intolerance, silent myocardial ischemia, arrhythmias, and increased mortality [11]. In patients with T2D, the prevalence of CAN may reach up to 60%, particularly in those with long-standing disease and concomitant microvascular complications [11].

Orthostatic hypotension (OH) is a clinically relevant manifestation that may occur in the setting of cardiovascular autonomic dysfunction and is defined as a sustained reduction in systolic blood pressure of ≥20 mmHg and/or diastolic blood pressure of ≥10 mmHg within three minutes of standing [12,13]. Approximately 20–25% of individuals with T2D experience OH, with higher prevalence reported among patients with longer diabetes duration, poorer glycemic control, and established microvascular disease [14]. OH may present with classical orthostatic symptoms such as dizziness and lightheadedness, but it can also occur asymptomatically or manifest as unexplained falls or syncope, complicating clinical recognition [13]. Importantly, bedside OH assessment is not equivalent to a comprehensive diagnosis of cardiovascular autonomic neuropathy (CAN), which generally requires formal autonomic reflex testing or heart-rate-based autonomic assessment. Nevertheless, OH remains a simple and clinically meaningful bedside marker of impaired orthostatic blood pressure regulation in older adults with diabetes.

Sudomotor dysfunction is a recognized component of diabetic autonomic neuropathy and reflects involvement of small unmyelinated C-fibers. Electrochemical skin conductance (ESC), assessed using Sudoscan, provides a rapid, non-invasive, and operator-independent measure of sudomotor function at the hands and feet. Lower ESC values indicate impaired sudomotor activity and small-fiber autonomic dysfunction. Prior studies have reported acceptable reproducibility and diagnostic utility for detecting distal symmetric polyneuropathy and autonomic impairment in diabetes [15,16].

Several studies have reported associations between abnormal Sudoscan parameters and established markers of diabetic microvascular disease, including peripheral neuropathy, nephropathy, and cardiovascular autonomic dysfunction [17,18]. Evidence regarding sudomotor dysfunction as a factor associated with OH remains limited, and Sudoscan is rarely integrated into predictive models for OH in patients with T2D.

The evidence linking balance dysfunction, falls, and autonomic impairment in T2D remains methodologically heterogeneous. Only a limited number of studies have explicitly examined autonomic manifestations as determinants of balance impairment, and the operationalization of functional outcomes varies substantially across the literature [8,19,20]. While performance-based balance measures and fear-of-falling constructs are increasingly incorporated into clinical studies, the independent contribution of orthostatic hypotension relative to peripheral neuropathy severity remains insufficiently clarified. This heterogeneity contributes to inconsistent findings and underscores the need for integrated clinical and autonomic assessment frameworks in older adults with T2D.

On this basis, we examined whether sudomotor dysfunction assessed by Sudoscan is associated with OH in patients with T2D, beyond the contribution of peripheral neuropathy severity and traditional clinical risk factors. Given the cross-sectional design, this hypothesis was tested only at the level of association, without implying temporality or causality.

### Aim

The primary aim of the present study was to identify clinical, neuropathic, and sudomotor factors associated with OH in patients with T2D, with Sudoscan-derived parameters evaluated as candidate autonomic markers rather than as diagnostic substitutes for formal CAN testing.

The secondary objectives were as follows:

- to examine the relationship between sudomotor function, peripheral neuropathy severity, and OH;
- to evaluate whether these associations persisted after adjustment for relevant clinical and metabolic covariates; and
- to explore the association between OH and balance performance and fear of falling as secondary, exploratory outcomes related to fall risk.

## 2. Materials and Methods

### 2.1. Study Design and Population

This was a cross-sectional, observational, single-center study of adults with T2D designed to identify clinical and autonomic factors associated with OH, with a particular focus on sudomotor dysfunction assessed by Sudoscan (Impeto Medical, Paris, France).

**Figure 1:**
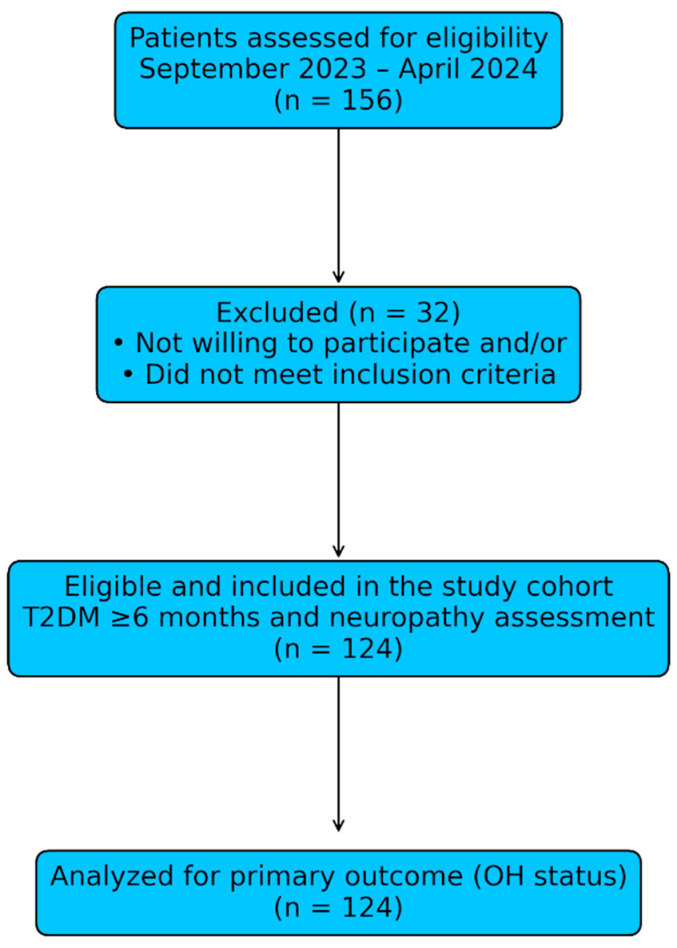
Study flow diagram.

The study included 124 adults with a confirmed diagnosis of T2D who were recruited between September 2023 and April 2024. Participants were eligible if they were aged 60 years or older, had a documented diabetes duration of at least 6 months, and were able to undergo clinical neuropathy evaluation, sudomotor testing, orthostatic blood pressure testing, and standardized functional assessments. The study flow diagram illustrating screening, exclusions, inclusion, and the analytic sample is presented in Figure 1.

Exclusion criteria comprise: (i) non-diabetic causes of neuropathy, screened through medical history, clinical evaluation, and review of available laboratory data (including vitamin B12 deficiency, chronic alcohol abuse, known neurotoxic chemotherapy exposure, and other documented peripheral neuropathies); (ii) a history of recent major cardiovascular events within the previous 6 months; (iii) neurological, vestibular, or musculoskeletal conditions significantly affecting balance (e.g., Parkinson’s disease, prior disabling stroke, and severe osteoarticular disease); (iv) use of medications with a major known impact on orthostatic blood pressure or balance (including centrally acting sedatives, antipsychotics, or high-dose tricyclic antidepressants); and (v) inability or refusal to provide written informed consent.

The study protocol and informed consent form were approved by the Ethics Committee of the ‘Victor Babes’ University of Medicine and Pharmacy in Timisoara, Romania (Approval No. 27; 5 July 2023), before participant recruitment began. All participants were recruited from the outpatient clinic of Brad Municipal Hospital, Hunedoara County, Romania. Given the single-center design, the findings should be interpreted with caution regarding external generalizability.

The study is reported in accordance with the Strengthening the Reporting of Observational Studies in Epidemiology (STROBE) Statement; the completed checklist is provided in the Supplementary Materials (Table S1) [21].

### 2.2. Baseline Clinical and Metabolic Assessment

At baseline, demographic characteristics, including age, sex, and place of residence, were recorded for all participants. Clinical assessment included the presence and severity of arterial hypertension (AH), as defined by contemporary European Society of Cardiology/European Society of Hypertension guidelines. Anthropometric evaluation included body mass index (BMI), calculated from measured height and weight.

Diabetes-related variables included T2D duration and glycemic control, assessed by HbA1c. Current glucose-lowering treatment and concomitant antihypertensive treatment were recorded from active medical records and confirmed during the study interview. Antihypertensive exposure was coded according to the medication classes being taken at the time of orthostatic testing. Because antihypertensive therapy may influence orthostatic blood pressure responses, these variables were examined as potential confounders in multivariable analyses. Dose, dosing schedule, and recent treatment changes were not consistently available.

### 2.3. Laboratory Measurements

Laboratory and metabolic parameters were extracted from clinical records and included serum magnesium (mg/dL), calcium (mg/dL), lipid profile components (total cholesterol, triglycerides, HDL-c, and LDL-c; mg/dL), inflammatory markers (C-reactive protein, mg/L, and erythrocyte sedimentation rate, mm/h), renal function indices (serum creatinine, mg/dL, and estimated glomerular filtration rate (eGFR), mL/min/1.73 m^2^), vitamin B12 (pg/mL), and urinary albumin-to-creatinine ratio (UACR, mg/g).

Laboratory values were extracted from routine clinical records and were used as clinically available variables rather than centrally repeated research assays. Renal parameters were retained because kidney involvement was clinically relevant to the study hypothesis: eGFR values were recorded as reported by the clinical laboratory using the CKD-EPI equation, and UACR was included a priori as an accessible marker of microvascular involvement potentially associated with autonomic dysfunction and OH. Laboratory measurements were obtained within 1 month of the autonomic and clinical assessments.

### 2.4. Autonomic Function Assessment

#### 2.4.1. Orthostatic Hypotension

Orthostatic blood pressure testing was performed after 10 min of supine rest, followed by repeated measurements at 1 and 3 min after active standing. OH was defined according to established diagnostic criteria as a decrease in systolic blood pressure of ≥20 mmHg and/or diastolic blood pressure of ≥10 mmHg within 3 min of standing. Participants were classified as having or not having OH, and OH status was treated as the primary dependent variable in explanatory models. Because Ewing battery testing, heart rate variability analysis, beat-to-beat monitoring, and tilt-table testing were not performed, OH was analyzed as a bedside clinical phenotype of orthostatic blood pressure dysregulation and not as a comprehensive diagnostic proxy for CAN.

#### 2.4.2. Sudomotor Function (Sudoscan)

Sudomotor function was assessed using SUDOSCAN (Impeto Medical, Paris, France), a non-invasive device that measures electrochemical skin conductance (ESC) as an objective marker of sudomotor activity mediated by small autonomic C-fibers. ESC was recorded at the palms and soles under standardized resting conditions. Lower ESC values indicate impaired sudomotor function. For each participant, ESC values for the hands and feet were recorded in microSiemens.

Sudomotor asymmetry indices were automatically calculated by the device as the relative difference between left- and right-sided ESC values. The Sudoscan-derived CAN and Nefro scores are composite proprietary scores generated from ESC measurements and demographic inputs; higher scores indicate greater autonomic or renal-risk estimates. Because their full derivation algorithms are proprietary and could not be independently recalculated, these composite scores were used only as exploratory device-derived variables and were not considered diagnostic endpoints.

Sudoscan testing was performed before orthostatic blood pressure testing under resting conditions and according to the standard operating procedure used in the clinic. To reduce pre-analytical variability, all participants underwent testing in a seated or standing-free resting state before the active standing maneuver. Ambient room temperature was not formally recorded; consequently, it could not be included as a covariate in multivariable analyses and is acknowledged as a potential source of residual measurement variability.

### 2.5. Peripheral Neuropathy Assessment

Diabetic neuropathy is assessed using the Michigan Neuropathy Screening Instrument (MNSI), which includes a symptom-based questionnaire and an objective clinical examination of the lower limbs, both applied according to validated protocols. It includes 2 components: MNSI-A (the questionnaire section) and MNSI-B (the examination section). Neuropathy severity is quantified using the corresponding MNSI scores, with higher values indicating greater neuropathic burden. MNSI is an internationally used and validated screening tool for DPN, with demonstrated internal consistency in validation studies (e.g., Cronbach’s α of 0.81 for the questionnaire section and 0.87 for the examination section) [20,22]. Because peripheral neuropathy and autonomic dysfunction frequently coexist in T2D, MNSI-A and MNSI-B are included as candidate covariates in models of OH.

### 2.6. Balance, Functional Mobility, and Fear-of-Falling Assessment

Balance performance, functional mobility, and fear of falling are evaluated as secondary (exploratory) outcomes using standardized and validated clinical instruments, including the Berg Balance Scale (BBS), the Timed Up and Go (TUG) test, the Falls Efficacy Scale–International (FES-I), and the Fear of Falling Questionnaire–Revised (FFQ-R). Perceived balance confidence and fear of falling were assessed using the FES-I and the FFQ-R, which capture complementary domains: FES-I primarily quantifies concern about falling during daily activities, whereas FFQ-R additionally evaluates emotional and behavioral responses related to fear of falling. Given the breadth of secondary outcomes, these analyses were considered exploratory and interpreted cautiously, given the potential for multiplicity and limited statistical power.

The BBS was used to assess static and dynamic balance through functional tasks, with lower scores indicating greater impairment. The scale demonstrates excellent internal consistency (Cronbach’s alpha ≈ 0.83–0.98) across diverse populations [23,24].

Functional mobility was assessed using the TUG test, which records the time required to stand, walk, turn, and sit; longer times indicate reduced mobility and higher fall risk. The TUG shows excellent test–retest and inter-rater reliability (ICC > 0.95) [25,26].

Static postural control was further evaluated using the Single-Leg Stance Test (SLS/SLST); shorter stance times reflect poorer balance. The test demonstrates good reliability in clinical populations (ICC ≈ 0.72) [27].

Depressive symptoms over the previous two weeks were assessed using the PHQ-9, a widely validated self-report instrument with excellent internal reliability (Cronbach’s alpha ≈ 0.89) [28]. Anxiety severity was measured using the GAD-7, which demonstrates similarly strong internal consistency (Cronbach’s alpha ≈ 0.92) [29].

Perceived balance confidence and fear of falling were assessed using the FES-I and the FFQ-R, which capture complementary domains. The FES-I primarily quantifies concern about falling during daily activities and shows excellent internal consistency (Cronbach’s alpha ≈ 0.96) [30,31]. The FFQ-R additionally evaluates emotional and behavioral responses related to fear of falling and demonstrates good-to-excellent reliability (Cronbach’s alpha ≈ 0.88–0.94) [32,33].

Global cognitive status was screened using the MoCA, a multi-domain instrument with good internal consistency (Cronbach’s alpha ≈ 0.84) in older adult populations [34].

### 2.7. Statistical Analysis

Statistical analysis was performed using MedCalc Statistical Software (version 23.4.5; MedCalc Software Ltd., Ostend, Belgium). Continuous variables were tested for normality using the Shapiro-Wilk test and are reported as the mean ± standard deviation or median [interquartile range], as appropriate, while categorical variables are presented as absolute frequencies and percentages. Group comparisons between participants with and without OH were conducted using the Student t-test or Mann–Whitney U test for continuous variables and the chi-square test for categorical variables. Given the number of univariate comparisons, these analyses were considered exploratory.

The primary outcome of the study was the presence of OH. Univariate analyses were first performed to explore associations between OH status and candidate explanatory variables, including clinical, metabolic, neuropathy-related, and sudomotor (Sudoscan-derived) parameters, using appropriate parametric or non-parametric tests.

Multivariable logistic regression was then used to model the probability of OH as the dependent variable. Candidate variables were prespecified a priori based on clinical relevance and existing evidence. Because of the limited number of OH events, the final model was kept parsimonious. Sudoscan-derived ESC parameters were examined as the main autonomic variables of interest. Because MNSI-A and MNSI-B capture related neuropathy constructs, collinearity was assessed and MNSI-B (the objective examination component) was prioritized in the primary model. Multicollinearity was evaluated using variance inflation factors. For continuous covariates, the assumption of logit linearity was assessed, and skewed variables, particularly UACR, were log-transformed before modeling. Antihypertensive medication classes were examined as potential confounders based on active treatment at the time of testing; however, dose, dosing schedule, treatment duration, and recent treatment changes were unavailable, and class-specific adjustment was not retained in the final parsimonious model because it would have reduced model stability relative to the number of events. No formal correction for multiple testing was applied because univariate and secondary analyses were exploratory; therefore, borderline *p*-values were interpreted cautiously. Model discrimination was quantified using the area under the receiver operating characteristic curve (AUC). Calibration was assessed using the Hosmer–Lemeshow goodness-of-fit test together with the Brier score. Because no external or bootstrap internal validation was performed, model performance metrics are reported as apparent performance and should not be interpreted as validated prediction accuracy.

Secondary exploratory analyses evaluated the associations of OH and sudomotor dysfunction with balance performance, functional mobility, and fear-of-falling outcomes using multivariable linear regression models for continuous outcomes (BBS, TUG, SLS, FES-I, and FFQ-R). When distributional assumptions were not fully met, appropriate transformations or robust approaches were considered. These analyses were regarded as hypothesis-generating.

## 3. Results

### 3.1. Baseline Characteristics

Baseline demographic, clinical, metabolic, neuropathy-related, and autonomic characteristics of the study cohort are summarized in Table 1. OH status was used to stratify participants in subsequent univariate and multivariable analyses.

The study cohort consists of 124 patients with T2D, with a mean age of 70.9 ± 5.9 years and a predominance of female patients (57.3%). Slightly more than half of the cohort (55.6%) resides in urban areas. At baseline, patients exhibit a high prevalence of overweight and obesity, with a mean BMI of 31.8 ± 4.4 kg/m^2^. These cardiometabolic features are clinically relevant, as both obesity and hypertension are known to influence autonomic regulation and orthostatic blood pressure responses. The median duration of diabetes is 9 years [5–14.5], and glycemic control is moderate, as reflected by a median HbA1c of 7.3% [6.5–8.15].

AH is highly prevalent in the cohort, with most patients classified as grade 2 hypertension (73.4%), while smaller proportions present with grade 3 hypertension (12.1%) or grade 1 hypertension (14.5%). Regarding antidiabetic therapy, metformin is the most frequently prescribed medication, used by 86.3% of patients, followed by sulphonylureas (61.3%), GLP-1 receptor agonists (30.6%), DPP-4 inhibitors (19.4%), and SGLT2 inhibitors (8.9%).

### 3.2. Univariate Associations with OH

#### 3.2.1. Clinical Metabolic and Psychological Variables

Univariate associations between OH and clinical and metabolic variables are presented in Table 2. These comparisons were exploratory and should be interpreted cautiously.

Patients with and without OH do not differ significantly in terms of age (*p* = 0.483) or BMI (*p* = 0.578). In contrast, diabetes duration is significantly longer in patients with OH than in those without (*p* < 0.001). Glycemic control showed a modest between-groups difference, with slightly higher HbA1c values in patients with OH (*p* = 0.047), although this finding should be interpreted cautiously given multiple testing. No statistically significant differences were observed between OH groups for psychological or cognitive measures, including PHQ-9 (*p* = 0.090), GAD-7 (*p* = 0.437), and MoCA scores (*p* = 0.692).

Laboratory and metabolic parameters, including serum electrolytes, lipid profile components, inflammatory markers, and renal function indices, were broadly comparable between patients with and without OH (all *p* > 0.05). In contrast, UACR was significantly higher in patients with OH (*p* = 0.010), suggesting greater microvascular involvement in this group. Antihypertensive medication classes were examined as potential confounders in multivariable modeling, but the available data did not support stable class-specific estimates in the final parsimonious model.

#### 3.2.2. Peripheral Neuropathy and Autonomic Variables (Sudoscan)

Univariate associations between OH and neuropathic variables are presented in Table 3. These analyses were exploratory and interpreted cautiously.

MNSI-A scores did not differ significantly between patients with and without OH (*p* = 0.185). In contrast, MNSI-B scores were higher in patients with OH (*p* = 0.028), while the total MNSI score showed a non-significant trend in the same direction (*p* = 0.068). Using established MNSI-B thresholds for probable diabetic peripheral neuropathy, the prevalence of DPN was high in both groups: 92.2% (47/51) in patients with OH and 84.9% (62/73) in those without OH, with no statistically significant difference between groups (χ^2^ = 0.873, *p* = 0.350).

Univariate comparisons of sudomotor function parameters according to OH status are presented below.

**Figure 2:**
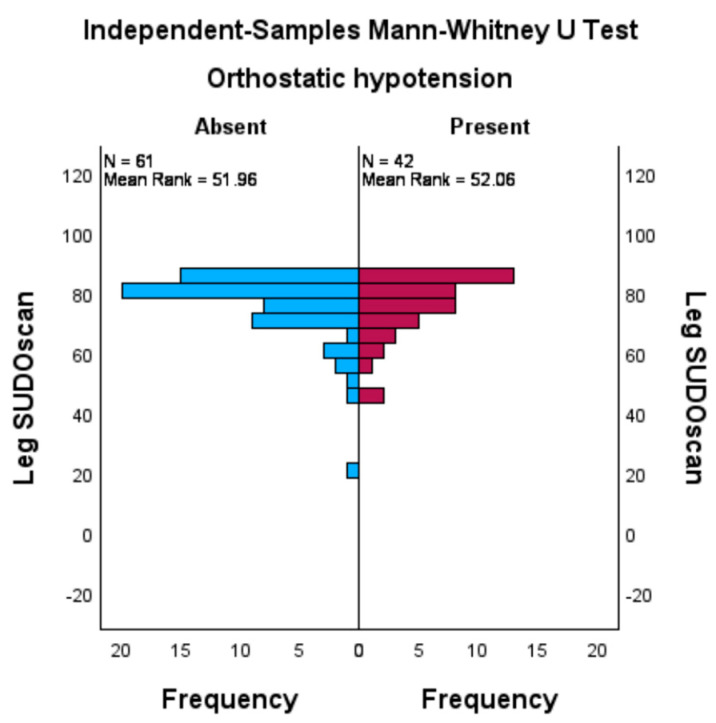
Distribution of lower-limb ESC measured by Sudoscan according to OH status.

Electrochemical skin conductance (ESC) measured at the feet showed similar distributions between groups, with no statistically significant difference (Mann–Whitney U = 1283.5, standardized test statistic = 0.017, *p* = 0.987), indicating comparable lower-limb sudomotor function across groups (Figure 2). Visual inspection of ESC distributions did not suggest relevant ceiling or floor effects that might have masked between-group differences.

**Figure 3:**
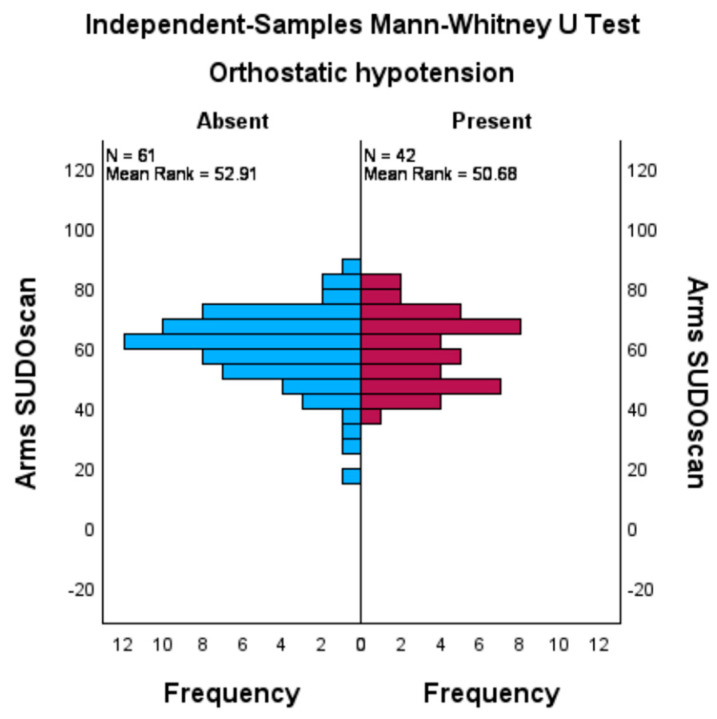
Distribution of upper-limb ESC measured by Sudoscan according to OH status.

Similarly, ESC values measured at the hands were comparable between groups, with no statistically significant difference according to OH status (Mann–Whitney U = 1225.5, standardized test statistic = −0.373, *p* = 0.709) (Figure 3).

**Figure 4:**
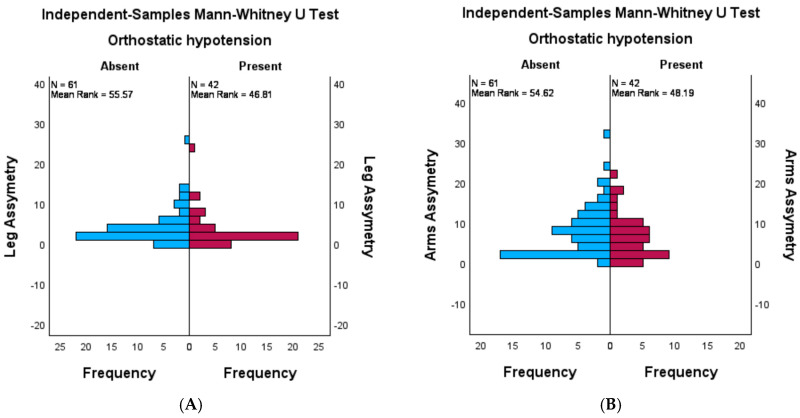
Comparison of Sudoscan-derived sudomotor asymmetry between patients with and without OH. (**A**) Leg asymmetry. (**B**) Arm asymmetry.

Measures of sudomotor asymmetry at the level of the feet and hands were also comparable between groups, with no significant differences observed for leg asymmetry (U = 1063.0, *p* = 0.138) or arm asymmetry (U = 1121.0, *p* = 0.281) (Figure 4A,B).

**Figure 5:**
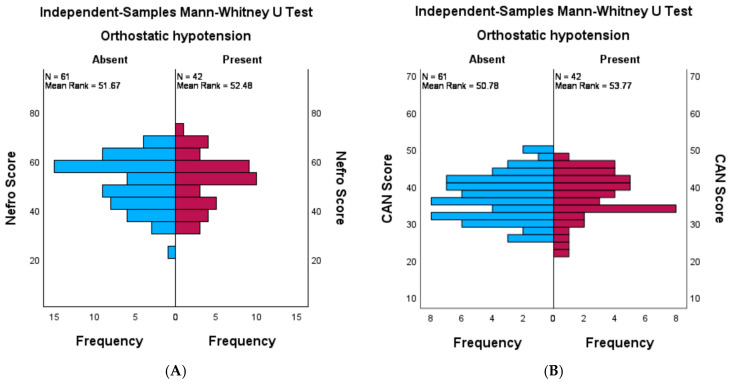
Comparison of composite autonomic dysfunction scores assessed by Sudoscan in patients with and without OH. (**A**) Nefro score; (**B**) CAN score.

Composite autonomic scores derived from Sudoscan (Nefro score and CAN score) did not differ significantly between patients with and without OH (Nefro score: U = 1301.0, *p* = 0.893; CAN score: U = 1355.5, *p* = 0.616) (Figure 5A,B).

### 3.3. Multivariable Factors Associated with OH

Although Sudoscan-derived parameters were evaluated because of their biological plausibility and relevance to the study question, they were not retained in the final multivariable model because they lacked significant or independent associations with OH in preliminary analyses. This negative result is central to the interpretation of the study.

Multivariable logistic regression was used to identify independent factors associated with OH using a parsimonious model that included diabetes duration, objective neuropathy severity (MNSI-B score), glycemic control (HbA1c), and log-transformed UACR. Antihypertensive medication classes were examined as potential confounders based on treatment at the time of testing; however, they were not retained in the final model because they were not materially associated with OH in this dataset and would have increased model instability relative to the number of events.

**Figure 6:**
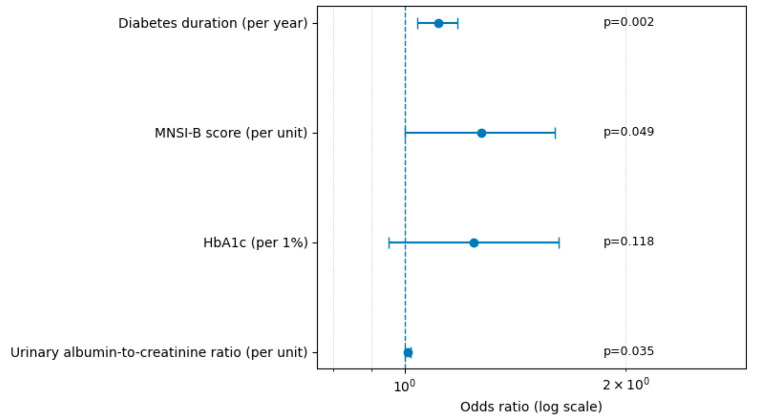
Forest plot of multivariable logistic regression factors associated with OH.

In the adjusted model (Figure 6), longer diabetes duration remained associated with OH, with each additional year of diabetes increasing the odds by approximately 11% (OR = 1.11, 95% CI 1.04–1.18; *p* = 0.002). Higher MNSI-B scores were also associated with OH (OR = 1.27, 95% CI 1.00–1.60; *p* = 0.049), although this borderline finding should be interpreted cautiously given the limited number of events and the exploratory nature of the analyses.

Log-transformed UACR remained independently associated with OH (OR = 1.01, 95% CI 1.00–1.02; *p* = 0.035), whereas HbA1c did not retain statistical significance after adjustment (OR = 1.24, 95% CI 0.95–1.62; *p* = 0.118).

The overall model (Table 4) showed moderate explanatory performance, with a Nagelkerke R^2^ of 0.257. Discrimination was acceptable, with an apparent area under the ROC curve of 0.787 (95% CI 0.673–0.885). Calibration results were mixed: the Hosmer–Lemeshow goodness-of-fit test yielded *p* = 0.025, suggesting imperfect calibration, whereas the Brier score was 0.184. Accordingly, the model should be interpreted as an explanatory model of association rather than a clinically ready prediction tool.

### 3.4. Secondary (Exploratory) Analyses: Balance and Fear of Falling

Secondary analyses examined associations among OH, balance performance, functional mobility, and fear-of-falling outcomes. These analyses were considered exploratory and interpreted cautiously.

Univariate comparisons of balance and functional outcomes according to OH status are summarized in Table 5. No statistically significant between-group differences were detected for BBS, TUG, or SLS in univariate comparisons (Table 5); effect sizes with confidence intervals are provided to contextualize the magnitude of these differences.

Similarly, fear-of-falling measures did not differ significantly according to OH status. Scores on the FES-I (*p* = 0.211) and the FFQ-R (*p* = 0.118) were comparable between patients with and without OH.

In prespecified exploratory multivariable analyses, each balance and fear-of-falling outcome (BBS, TUG, SLS, FES-I, and FFQ-R) was modeled as a continuous dependent variable using multivariable linear regression. To enhance transparency, the full results of these models are provided in Supplementary Table S2. All models were adjusted for age, diabetes duration, MNSI-B, PHQ-9, GAD-7, and Sudoscan ESC measured at the feet. These analyses were hypothesis-generating and were not intended to support causal inference.

## 4. Discussion

In this cross-sectional cohort of older adults with T2D, OH was associated with longer diabetes duration, greater objective peripheral neuropathy severity, and higher UACR. These findings should be interpreted strictly as associations. They suggest that OH identifies patients with a greater cumulative burden of neuropathic and microvascular disease, but they do not establish temporal sequence, causality, or mechanistic progression.

Sudoscan-derived parameters were not associated with OH in univariate analyses and did not contribute independently in multivariable modeling. This is an important negative finding. It indicates that, in the present cohort, ESC-based sudomotor measures and proprietary Sudoscan composite scores were not sufficient to identify patients with bedside-defined OH. Therefore, the present data do not support the use of Sudoscan as a stand-alone surrogate marker for OH or as a replacement for direct orthostatic blood pressure assessment.

These results are directionally consistent with previous work showing that OH is more common in patients with longer diabetes duration and more advanced neuropathy [35,36,37,38,39]. However, because formal cardiovascular autonomic reflex tests were not performed, the present study should not be interpreted as a comprehensive CAN study. OH was used as a clinically relevant bedside phenotype of impaired orthostatic blood pressure regulation, while CAN remains a broader construct requiring more extensive autonomic testing.

The association between OH and MNSI-B supports the coexistence of orthostatic blood pressure dysregulation with objective peripheral neuropathy in older adults with T2D. Nevertheless, the *p*-value for MNSI-B was borderline, and the estimate should be considered hypothesis-generating rather than definitive. Similarly, the association between OH and UACR is biologically plausible because albuminuria is an accessible marker of microvascular and endothelial injury, but the cross-sectional design does not allow conclusions about whether albuminuria contributes to OH, results from shared vascular pathways, or simply marks a more advanced diabetes phenotype.

The absence of significant univariate differences in balance, mobility, and fear-of-falling outcomes according to OH status also requires a conservative interpretation. Postural instability in diabetes is multifactorial and may depend on peripheral neuropathy severity, muscle performance, visual status, vestibular function, psychological factors, comorbidity burden, and medication exposure. In the present sample, OH alone did not differentiate balance or fear-of-falling measures, and the secondary analyses should be regarded as exploratory and underpowered for definitive functional conclusions.

### 4.1. Clinical Implications

From a practical clinical perspective, the findings support routine orthostatic blood pressure measurement in older adults with T2D, particularly in those with long diabetes duration, objective neuropathy, albuminuria, hypertension, or polypharmacy. The results also emphasize that orthostatic assessment should complement, not replace, peripheral neuropathy evaluation and kidney complication screening. When OH is identified, clinicians should consider medication review, hydration and volume-status assessment, evaluation for symptoms or falls, and individualized fall-risk prevention. At the same time, the present findings do not justify using the regression model as a bedside risk score, because calibration was imperfect and neither internal nor external validation was performed.

The findings also have implications for the interpretation of Sudoscan in clinical practice. Sudoscan may remain useful as part of broader small-fiber or sudomotor assessment, but a normal or abnormal ESC value should not be assumed to exclude or confirm OH. Direct orthostatic blood pressure testing remains necessary when OH is clinically suspected.

### 4.2. Limitations and Future Directions

Several limitations should be acknowledged. First, the cross-sectional design precludes causal inference, temporal sequencing, and progression-based interpretations. Second, comprehensive cardiovascular autonomic reflex testing was not performed, so OH should not be equated with a full diagnosis of CAN. Third, antihypertensive treatment was captured only at the class level at the time of testing; dose, dosing schedule, treatment duration, recent changes, and adherence were not consistently available. Therefore, residual confounding by medication exposure and polypharmacy cannot be excluded. Fourth, the multivariable model included only 51 OH events, showed imperfect calibration, and was not internally or externally validated; it should therefore be interpreted as explanatory and hypothesis-generating rather than predictive. Fifth, multiple univariate and secondary analyses were performed without multiplicity correction, and borderline associations should be interpreted cautiously. Sixth, balance and fear-of-falling outcomes were exploratory and may have been underpowered. Seventh, the study was single-center, clinic-based, restricted to adults aged 60 years or older, and included a population with a high prevalence of neuropathy; consequently, external generalizability to younger adults, primary-care populations, or patients with earlier T2D may be limited. Finally, ambient temperature during Sudoscan testing was not formally recorded and could not be included as a confounder, and the proprietary nature of Sudoscan composite scores limits independent reproducibility.

Prospective longitudinal studies incorporating Sudoscan alongside standardized cardiovascular autonomic testing are warranted to clarify whether sudomotor abnormalities precede, accompany, or remain independent from the development of OH. Larger multicenter cohorts with detailed medication data and prespecified validation strategies should enable better-adjusted models and determine whether any clinically useful prediction framework can be developed.

## 5. Conclusions

In this cross-sectional cohort of older adults with T2D, OH was associated with longer diabetes duration, higher objective neuropathy severity, and greater albuminuria. These findings identify a clinical profile associated with OH but do not establish causality or disease progression.

Sudoscan-derived measures were not independently associated with OH. Therefore, Sudoscan should not be interpreted as a stand-alone marker for OH in this setting, and direct orthostatic blood pressure assessment remains necessary.

OH was not associated with significant univariate differences in balance or fear-of-falling measures in this sample. These exploratory findings should be interpreted cautiously, particularly given the limited sample size, residual medication confounding, and absence of formal autonomic reflex testing.

## Figures and Tables

**Table 1:** Baseline characteristics of the study cohort.

Variable	Total Cohort (n = 124)
Age, years	70.9 ± 5.9
Male	53 (42.7%)
Female	71 (57.3%)
Residence	
Urban	69 (55.6%)
Rural	55 (44.4%)
BMI, kg/m^2^	31.8 ± 4.4
Diabetes duration, years	9 [5–14.5]
HbA1c, %	7.3 [6.5–8.15]
Arterial hypertension	
Grade 1	18 (14.5%)
Grade 2	91 (73.4%)
Grade 3	15 (12.1%)
Antidiabetic treatment	
Metformin	107 (86.3%)
Sulphonylurea	76 (61.3%)
GLP-1 receptor agonist	38 (30.6%)
DPP-4 inhibitor	24 (19.4%)
SGLT2 inhibitor	11 (8.9%)

Continuous variables are presented as the mean ± SD when normally distributed and as median [interquartile range] when non-normally distributed, based on the Shapiro-Wilk test. Antidiabetic medication categories are not mutually exclusive; percentages reflect the proportion of patients receiving each drug class, alone or in combination.

**Table 2:** Clinical and metabolic variables according to OH status.

Variable	OH Absent (n = 73)	OH Present (n = 51)	*p*-Value *
Age, years	71.0 [68.0–75.0]	70.0 [66.5–75.0]	0.483
BMI, kg/m^2^	31.3 [28.4–34.3]	32.3 [28.9–34.6]	0.578
Diabetes duration, years	7.0 [4.0–12.0]	13.0 [9.0–17.0]	**<0.001**
HbA1c, %	7.2 [6.5–7.8]	7.5 [6.8–8.9]	**0.047**
PHQ-9 score	7.0 [4.0–11.0]	9.0 [5.0–12.0]	0.090
GAD-7 score	6.0 [2.0–11.0]	8.0 [2.0–11.5]	0.437
MoCA score	23.0 [19.0–25.0]	23.0 [19.0–26.0]	0.692
Magnesium, mg/dL	1.8 [1.6–1.9]	1.8 [1.6–1.9]	0.987
Calcium, mg/dL	9.6 [9.3–9.9]	9.5 [9.3–9.8]	0.843
Total cholesterol, mg/dL	175.0 [154.0–202.0]	176.0 [146.5–217.5]	0.537
Triglycerides, mg/dL	137.0 [99.0–189.0]	148.0 [107.0–195.0]	0.361
HDL-c, mg/dL	43.0 [38.0–52.0]	44.0 [35.5–50.8]	0.378
LDL-c, mg/dL	99.0 [76.2–123.8]	102.5 [71.4–134.3]	0.732
C-reactive protein, mg/L	2.6 [1.2–6.1]	2.7 [1.3–5.6]	0.802
ESR, mm/h	22.0 [14.0–30.0]	20.0 [12.0–34.5]	0.925
Creatinine, mg/dL	0.9 [0.7–1.1]	0.8 [0.7–1.0]	0.925
eGFR, mL/min/1.73 m^2^	77.2 [62.2–89.5]	78.2 [61.6–87.6]	0.925
UACR, mg/g	11.3 [7.2–30.3]	19.6 [9.3–54.5]	**0.010**
Vitamin B12, pg/mL	294.5 [218.5–407.8]	267.5 [205.2–416.0]	0.998

* Data are compared between groups using the Mann–Whitney U test. OH, orthostatic hypotension; BMI, body mass index; HbA1c, glycated hemoglobin; PHQ-9, Patient Health Questionnaire-9; GAD-7, Generalized Anxiety Disorder-7; MoCA, Montreal Cognitive Assessment; HDL-c, high-density lipoprotein cholesterol; LDL-c, low-density lipoprotein cholesterol; ESR, erythrocyte sedimentation rate; eGFR, estimated glomerular filtration rate; UACR, urinary albumin-to-creatinine ratio. *p*-values in bold represents statistically significant differences.

**Table 3:** Neuropathic variables according to OH status.

Variable	OH Absent (n = 73)	OH Present (n = 51)	*p*-Value *
MNSI-A score	4.0 [2.0–5.0]	5.0 [3.0–5.0]	0.185
MNSI-B score	5.0 [4.0–6.0]	5.5 [4.5–7.0]	**0.028**
Total MNSI score	8.5 [5.5–12.0]	10.0 [8.0–12.0]	0.068

* Group comparisons were performed using the Mann–Whitney U test. Abbreviations are listed at the end of the manuscript. *p*-values in bold represent statistically significant differences.

**Table 4:** Multivariable logistic regression model for OH.

Associated Factors	B (SE)	Wald χ^2^	OR (95% CI)	*p*-Value
Diabetes duration (years)	0.100 (0.032)	9.637	1.11 (1.04–1.18)	0.002
MNSI-B score	0.235 (0.119)	3.876	1.27 (1.00–1.60)	0.049
HbA1c (%)	0.215 (0.137)	2.446	1.24 (0.95–1.62)	0.118
Ln(UACR)	0.008 (0.004)	4.457	1.01 (1.00–1.02)	0.035
Constant	−4.501 (1.285)	12.271	-	<0.001

B, regression coefficient; SE, standard error. Additional abbreviations are listed at the end of the manuscript.

**Table 5:** Balance, functional mobility, and fear-of-falling measures according to OH status.

Variable	OH Absent (n = 73)	OH Present (n = 51)	*p*-Value *	Hodges–Lehmann Median Difference (95% CI)
BBS score	43.0 [38.0–48.0]	42.0 [35.5–50.0]	0.540	−1.0 (−5.00 to 3.00)
SLS (s)	6.9 [2.4–13.2]	3.9 [1.4–10.6]	0.104	−1.5 (−3.70 to 0.30)
TUG (s)	11.5 [10.0–13.0]	12.6 [10.3–14.3]	0.230	0.7 (−0.40 to 1.89)
FES-I score	27.0 [20.0–39.0]	32.0 [22.5–40.0]	0.211	4.0 (0.00 to 8.00)
FFQ-R score	14.0 [4.0–30.0]	23.0 [9.5–33.5]	0.118	5.0 (−1.00 to 10.00)

* Non-parametric comparisons were conducted using the Mann–Whitney U test. Effect sizes are reported as Hodges–Lehmann median difference with 95% confidence intervals.

## Data Availability

The data are not publicly available due to local privacy and data protection regulations.

## Supplementary Materials

The following supporting information can be downloaded at: https://www.mdpi.com/article/10.3390/healthcare14111515/s1. Table S1. STROBE checklist, Table S2. Multivariable linear regression analyses for balance and fear-of-falling outcomes.

## Abbreviations

The following abbreviations are used in this manuscript:

ADA	American Diabetes Association
AH	Arterial hypertension
BBS	Berg Balance Scale
BMI	Body mass index
CAN	Cardiovascular autonomic neuropathy
CI	Confidence interval
CRP	C-Reactive Protein
DAN	Diabetic autonomic neuropathy
DN	Diabetic neuropathy
DPN	Diabetic peripheral neuropathy
eGFR	Estimated glomerular filtration rate
ESC	Electrochemical skin conductance
ESC/ESH	European Society of Cardiology/European Society of Hypertension
FES-I	Falls Efficacy Scale–International
FFQ-R	Fear of Falling Questionnaire–Revised
GAD-7	Generalized Anxiety Disorder-7
HbA1c	Glycated hemoglobin
HDL-c	High-Density Lipoprotein Cholesterol
ICC	Intraclass correlation coefficient
IDMS	Isotope dilution mass spectrometry
LDL-c	Low-Density Lipoprotein Cholesterol
MNSI	Michigan Neuropathy Screening Instrument
MoCA	Montreal Cognitive Assessment
OH	Orthostatic hypotension
OR	Odds Ratio
PHQ-9	Patient Health Questionnaire-9
UACR	Urinary Albumin-to-Creatinine Ratio
SD	Standard deviation
SLS	Single-Leg Stance Test
T2D	Type 2 diabetes
TUG	Timed Up and Go Test
