# Supplementary material for: Clinical, Neuropathic, and Sudomotor Correlates of Orthostatic Hypotension in Type 2 Diabetes: A Cross-Sectional Study
Source: Healthcare (Basel). 2026 May 29;14(11):1515. doi: 10.3390/healthcare14111515 (PMC13256756; doi:10.3390/healthcare14111515)
Supplement: Supplementary file 1 [file healthcare-14-01515-s001.zip › healthcare-4284064-supplementary.pdf]

Table S1. STROBE checklist

| | Item No | Recommendation | Reported (page/section) |
|---|---|---|---|
| **Title and abstract** | 1 | (*a*) Indicate the study's design with a commonly used term in the title or the abstract | Yes — Title includes "Cross-Sectional Study." |
| | | (*b*) Provide in the abstract an informative and balanced summary of what was done and what was found | Yes — Structured abstract provided |
| **Introduction** | | | |
| Background/rationale | 2 | Explain the scientific background and rationale for the investigation being reported | Yes — Introduction |
| Objectives | 3 | State-specific objectives, including any prespecified hypotheses | Yes – Section 1.1 Aim |
| **Methods** | | | |
| Study design | 4 | Present key elements of study design early in the paper | Yes — Section 2.1 |
| Setting | 5 | Describe the setting, locations, and relevant dates, including periods of recruitment, exposure, follow-up, and data collection | Yes — recruitment period specified |
| Participants | 6 | (*a*) *Cohort study*—Give the eligibility criteria, and the sources and methods of selection of participants. Describe methods of follow-up<br>*Case-control study*—Give the eligibility criteria, and the sources and methods of case ascertainment and control selection. Give the rationale for the choice of cases and controls<br>*Cross-sectional study*—Give the eligibility criteria, and the sources and methods of selection of participants | Yes — inclusion/exclusion listed |
| | | (*b*) *Cohort study*—For matched studies, give matching criteria and number of exposed and unexposed<br>*Case-control study*—For matched studies, give matching criteria and the number of controls per case | Not applicable |
| Variables | 7 | Clearly define all outcomes, exposures, predictors, potential confounders, and effect modifiers. Give diagnostic criteria, if applicable | Yes — OH, ESC, MNSI defined |
| Data sources/ measurement | 8* | For each variable of interest, give sources of data and details of methods of assessment (measurement). Describe comparability of assessment methods if there is more than one group | Yes — clinical and device methods described |
| Bias | 9 | Describe any efforts to address potential sources of bias | Partially — discussed in limitations |
| Study size | 10 | Explain how the study size was arrived at | Implicit — cohort described |
| Quantitative variables | 11 | Explain how quantitative variables were handled in the analyses. If applicable, describe which groupings were chosen and why | Yes — statistical section |
| Statistical methods | 12 | (*a*) Describe all statistical methods, including those used to control for confounding | Yes — detailed |

| | | | |
|---|---|---|---|
| | | (*b*) Describe any methods used to examine subgroups and interactions | Yes — exploratory analyses |
| | | (*c*) Explain how missing data were addressed | Not explicitly applicable/none reported |
| | | (*d*) *Cohort study*—If applicable, explain how loss to follow-up was addressed<br>*Case-control study*—If applicable, explain how matching of cases and controls was addressed<br>*Cross-sectional study*—If applicable, describe analytical methods taking account of sampling strategy | Not applicable |
| | | (*e*) Describe any sensitivity analyses | Yes — MedCalc specified |

| **Results** | | | **Reported (page/section)** |
|---|---|---|---|
| Participants | 13* | (a) Report numbers of individuals at each stage of study—eg numbers potentially eligible, examined for eligibility, confirmed eligible, included in the study, completing follow-up, and analysed | Yes — n = 124 |
| | | (b) Give reasons for non-participation at each stage | Non applicable |
| | | (c) Consider use of a flow diagram | Not included |
| Descriptive data | 14* | (a) Give characteristics of study participants (eg demographic, clinical, social) and information on exposures and potential confounders | Yes — Table 1 |
| | | (b) Indicate number of participants with missing data for each variable of interest | Not present |
| | | (c) *Cohort study*—Summarise follow-up time (eg, average and total amount) | Not present |
| Outcome data | 15* | *Cohort study*—Report numbers of outcome events or summary measures over time | Yes — OH prevalence and analyses |
| | | *Case-control study*—Report numbers in each exposure category, or summary measures of exposure | Yes |
| | | *Cross-sectional study*—Report numbers of outcome events or summary measures | Yes |
| Main results | 16 | (*a*) Give unadjusted estimates and, if applicable, confounder-adjusted estimates and their precision (eg, 95% confidence interval). Make clear which confounders were adjusted for and why they were included | Yes — multivariable model |
| | | (*b*) Report category boundaries when continuous variables were categorized | Yes, where applicable |
| | | (*c*) If relevant, consider translating estimates of relative risk into absolute risk for a meaningful time period | Not applicable |
| Other analyses | 17 | Report other analyses done—eg analyses of subgroups and interactions, and sensitivity analyses | Yes — secondary exploratory |
| **Discussion** | | | |
| Key results | 18 | Summarise key results with reference to study objectives | Yes — opening paragraph |
| Limitations | 19 | Discuss limitations of the study, taking into account sources of potential bias or imprecision. Discuss both direction and magnitude of any potential bias | Yes — dedicated section |

| | | | |
|---|---|---|---|
| Interpretation | 20 | Give a cautious overall interpretation of results considering objectives, limitations, multiplicity of analyses, results from similar studies, and other relevant evidence | Yes — integrated discussion |
| Generalisability | 21 | Discuss the generalisability (external validity) of the study results | Yes — single-center noted |
| **Other information** | | | |
| Funding | 22 | Give the source of funding and the role of the funders for the present study and, if applicable, for the original study on which the present article is based | Yes — Funding section |

Table S2. Multivariable linear regression analyses for balance and fear-of-falling outcomes

| ***Outcome*** | ***β (SE) for OH*** | ***95% CI*** | ***p-value*** | ***N*** | ***Covariates*** |
|---|---|---|---|---|---|
| **BBS** | 0.58 (1.37) | −2.15 to 3.30 | 0.675 | 103 | age, diabetes duration, MNSI-B, PHQ-9, GAD-7, ESC feet |
| **TUG** | 0.10 (0.63) | −1.15 to 1.34 | 0.877 | 103 | age, diabetes duration, MNSI-B, PHQ-9, GAD-7, ESC feet |
| **SLS** | −3.00 (2.06) | −7.08 to 1.08 | 0.148 | 103 | age, diabetes duration, MNSI-B, PHQ-9, GAD-7, ESC feet |
| **FES-I** | 0.39 (1.82) | −3.23 to 4.01 | 0.830 | 103 | age, diabetes duration, MNSI-B, PHQ-9, GAD-7, ESC feet |
| **FFQ-R** | 1.80 (2.60) | −3.36 to 6.95 | 0.490 | 103 | age, diabetes duration, MNSI-B, PHQ-9, GAD-7, ESC feet |
